# Premature Death as a Normative Concept

**DOI:** 10.1007/s10728-023-00471-x

**Published:** 2024-01-26

**Authors:** Preben Sørheim, Mathias Barra, Ole Frithjof Norheim, Espen Gamlund, Carl Tollef Solberg

**Affiliations:** 1 Department of Philosophy, Faculty of Humanities, University of Bergen, Sydnesplassen 1213, Postbox 7805, 5020 Bergen, Norway; 2 The Health Services Research Unit—HØKH, Akershus University Hospital, Lørenskog, Norway; 3 Department of Global Public Health and Primary Care, Faculty of Medicine, Bergen Centre for Ethics and Priority Setting—BCEPS, University of Bergen, Bergen, Norway; 4 Harvard TH Chan School of Public Health, Harvard University, Boston, USA; 5 Faculty of Medicine, Centre for Medical Ethics (CME), Institute of Health and Society, University of Oslo, Oslo, Norway

**Keywords:** Death, Epidemiology, Ethics, Public policy, Philosophy

## Abstract

The practical goal of preventing premature death seems uncontroversial. But the term ‘premature death’ is vague with several, sometimes conflicting definitions. This ambiguity results in several conceptions with which not all will agree. Moreover, the normative rationale behind the goal of preventing premature deaths is masked by the operational definition of existing measures. In this article, we argue that ‘premature death’ should be recognized as a normative concept. We propose that normative theories should be used to justify measures of premature death to provide them with normative validity and public legitimacy.

## Introduction

The prevention of premature deaths is often put forward as an important goal in global health initiatives. Most prominent, perhaps, is the UN Sustainable Development Goal’s target 3.4., which aims to significantly reduce premature mortality^1^ from non-communicable diseases [1]. Another notable example is the Global Burden of Disease (GBD) study, which attempts to quantify the combined burden of premature death and disability in order to guide funding, policies, and interventions [2, 3]. Moreover, many countries and international organizations employ their own measures of premature death in a variety of settings, particularly in public health [4].

Despite its ubiquity in health policy documents, the concept of premature death has received little theoretical attention.^2^ One explanation might be its innocuous appearance: preventing premature deaths involves giving priority to the worse-off—in which the short-lived are, plausibly, included [7]—thus forming a practical goal that proponents of diverging views about distributive justice can agree with [8]. Here, we argue that it is unclear what this agreement is about.

For instance, the UN simply defines *deaths before the age of 70 years* as premature. The GBD study, on the other hand, quantifies the total years of life lost (YLL) in a population. YLLs measure the gap between the age at death and the remaining life expectancy for that age. The GBD study's approach relies on the assumption that every death is premature. As we shall argue, there are different conceptions of *premature death* that underlie different efforts to prevent it, and these cannot all be sustained by the same normative considerations.

Moreover, existing measures seem to mask the normative motivation behind such efforts. The measures have operational definitions, such as *death before the age of 75 years*, that introduce features—such as the implicit sharp contrast between death at 74 and 75 years—which seems hard to justify if not outright normatively insignificant. At the same time, these measures are important parts of normatively motivated efforts that can end up as controversial when many initiatives to prevent premature deaths will, directly or indirectly, prioritize younger over older persons. Despite broad support for some such efforts (see, e.g., [9]), the issue itself is contentious. Concrete proposals (e.g., [10]) are thus often contested on the grounds of ageism (e.g., [11]).

The consensus behind the prevention of premature deaths, as it is defined by its common measures, might therefore be illusory. Quantitative measures may take on a life and a meaning of their own as they are increasingly used, particularly in institutional contexts. This process might resemble Goodhart’s law, which states that “when a measure becomes a target, it ceases to be a good measure.” However, while premature death measures might not be good measures, they might still be good targets.

Thus, the aim of this article is to carve out a space for premature death measures which recognizes that their primary function is as normative goals. We shall argue that the problem with premature mortality measures—as they are currently used in epidemiology and related disciplines—is their low normative validity.^3^ We propose that their normative validity can be improved by additional justification from normative theories that are adapted to the contexts in which these measures occur. We call these normative theories *normative background theories* because we intend them to remain in the background for exogenous support.

Our proposal is thus to keep the existing measures largely as they are but also to recognize that their referent—premature death—is a normative concept throughout the process of conceptualization, definition, and measurement. It follows that the choice of a particular measure of premature death must be normatively justified, and care must be given to ensure that the values embedded in the chosen measure of premature death align with those of the broader context within which it is to be employed,

The article proceeds as follows: Sect. “Three conceptions of premature death” categorizes common types of measures by their conception of premature death. In Sect. “Conceptual Issues”, we discuss some conceptual issues of these categories. We then argue that improved normative justification of the measures in question may alleviate these conceptual issues. In Sect. “Normative background theories”, we show how normative background theories, which provide a link between measures and legitimate normative goals, can buttress the normative validity of the measures. We suggest and review four candidate theories. We conclude that the most common types of measures of premature death can be supported by legitimate normative goals.

**Table 1:**
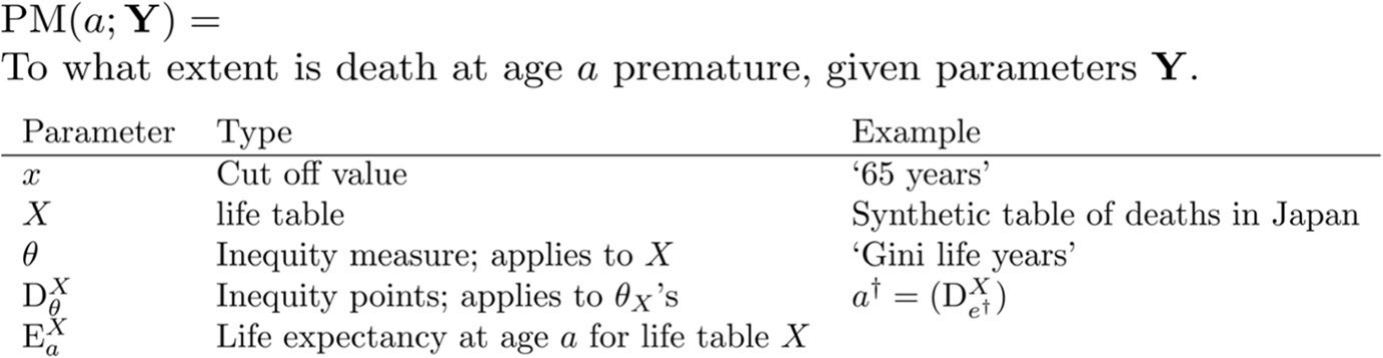
Explanations to Fig. 1.

## Three Conceptions of Premature Death

**Fig. 1:**
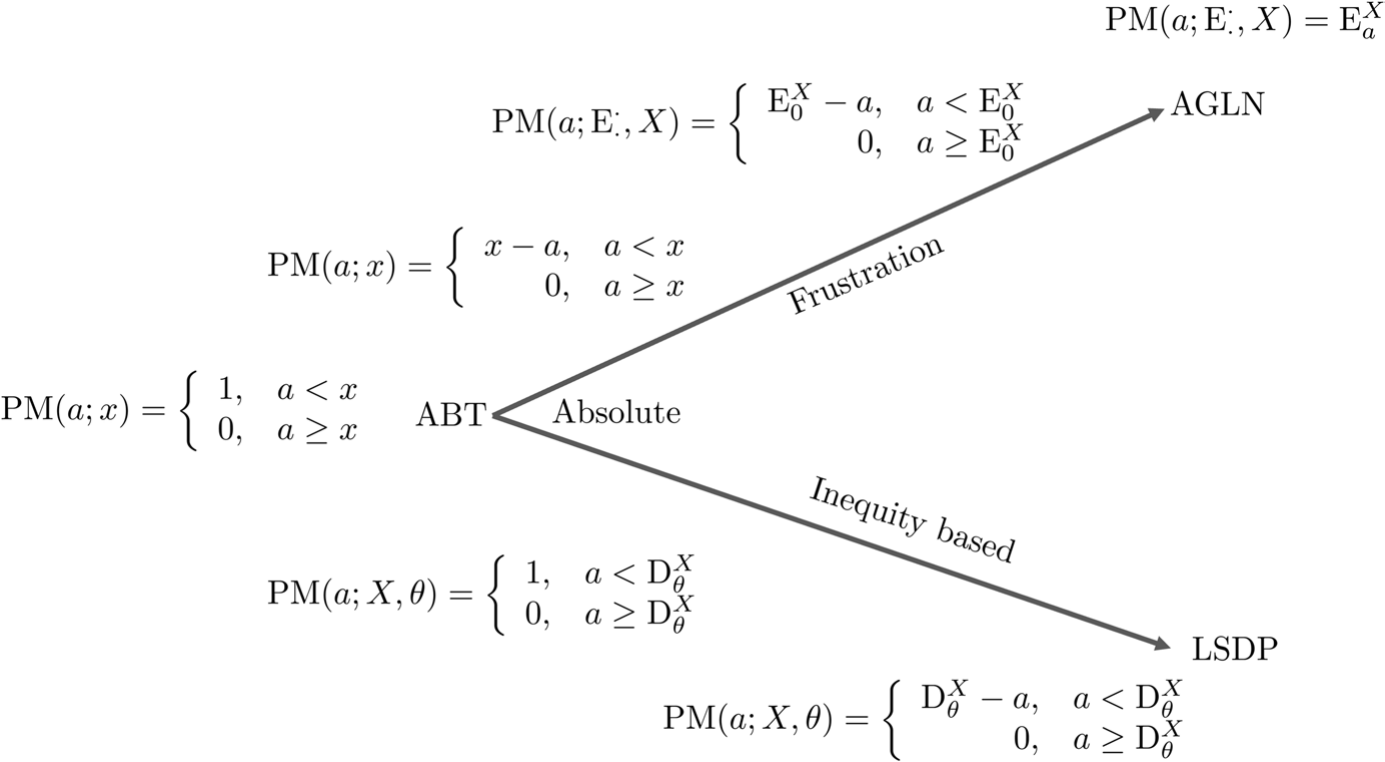
The figure illustrates the two directions along which one develops the ABT into more 'sophisticated' PM-measures. These modifications fall into either or both of two broad categories: (1) mitigating critiques about the implausibility of dichotomizing thresholds—making prematureness a graded concept instead—and, (2) about the arbitrariness of the point that separates premature deaths from non-premature ones—attempting to give a better justification of the cut-off value

Many measures are purported to track premature death (for an overview, see Table 2). We will limit our focus to measures that take chronological age to be the ‘currency’ of premature death. We categorize different types of these measures by how they classify deaths as premature. This results in three categories that then correspond to distinct conceptions of premature death (see Fig. 1). First, there are *age-based thresholds* (ABTs) which classify a death as premature if it occurs under a pre-defined, absolute threshold. Second, there are *age-group longevity norms* (AGLNs) which quantify premature death as the gap between the time of death of an individual and the expected remaining lifetime in some chosen reference population. Third, there are *lifespan disparity points* (LSDPs) which classify a death as premature if its occurrence increases a population’s lifespan disparity.

ABTs posit a threshold *x*. Deaths under the threshold are considered premature, which implies that deaths over the threshold are not. ABTs thus function as *classifiers* which are parameterized by the threshold value *x*. Once this value is given, the ABT provides a true-or-false-answer (1 vs. 0) to the question of whether a death is premature. Stated formally,

$${\text{PM}}\left( {a;\;x} \right) = \left\{ {\begin{array}{*{20}l} {1, } \hfill & {a < x} \hfill \\ {0, } \hfill & {a \ge x} \hfill \\ \end{array} ,} \right.$$

where PM is the generic acronym for any premature death measure. While *x* is a priori arbitrary, it will usually be informed by other value judgements that are thought to be relevant to distinguish between early and timely, or late, deaths, or something similar, for the context to which it is applied. For instance, the UN definition currently uses death before the age of 70 years as a definition of premature death [10], which is close to the global average life expectancy at birth [13].

AGLNs implement a different approach. They calculate, for instance, the remaining life expectancy for different age groups, which then constitutes norms for how many life-years are lost when death occurs at a given age. These norms depend on a choice of reference population (*X*). AGLNs thus quantify the gap in longevity between an actual death and the death of those in the reference population. The use of an AGLNs implies that 'everybody dies prematurely' [14], and take the form ${\text{PM}}\left( {a;\;{\text{E}},X} \right) = {\text{E}}_{a}^{X}$, where ${\mathrm{E}}_{a}>0$ is a measure of remaining expected life-years among those who have survived until age *a*.^4^ Stated formally,

$${\text{PM}}\left( {a;\;{\text{E}},X} \right) = {\text{E}}_{a}^{X}$$

In principle, one can choose between several different reference populations (e.g., the estimated life expectancy of a cohort as it ages or an 'ideal population' in very good health). A prominent example of an AGLNs is *years of life lost* (YLLs), which quantifies the burden of premature death in the *disability-adjusted life year* (DALY).

A third type of measure of premature death is the *lifespan disparity points* (LSDPs). An LSDP is a markedly different conception from ABTs and AGLNs. Like an AGLN, the LSDP are based on the distribution of age at death in a population. An LSDP is an ABT, but the motivation behind an LSDP is different. An LSDP sets the threshold value where it separates deaths that increase lifespan disparity from those that decrease lifespan disparity. The parameters to an LSDP are thus both a distribution of deaths *X* and a measure of inequality $\theta$. Stated formally,

$${\text{PM}}\left( {a;\;{\rm X},\theta } \right) = \left\{ {\begin{array}{*{20}l} {D_{\theta }^{X} - a,} \hfill & {a < \theta_{X}^{\dag } } \hfill \\ {0,} \hfill & {a \ge \theta_{X}^{\dag } } \hfill \\ \end{array} } \right.$$

One example is the demographic measure of lifespan disparity *e*^*†*^ (*e-dagger*), which measures average number of life years lost. Under most circumstances, this disparity measure implies a threshold value *a*^*†*^ for a population [15]. Averting deaths below *a*^*†*^ decreases *e*^*†*^, whereas averting deaths above the threshold increases disparity. This disparity point thus becomes a moving target contingent on the development of a population’s longevity over time (this, of course, is also true for the AGLN﻿) (Fig. 1 and Table 1)

### Parity Between Conceptions

We claim that the three conceptions above are roughly *on a par*: neither is more plausible as a conception of premature death than the others, nor are the measures contained in any category more plausible either [16]. Our primary reason is that the term 'premature death' lacks a semantic core that indicates any specific conception. Take, for instance, the main contention between the conceptions of premature death in ABTs and AGLNs. The former suggests that death above a certain point is no longer premature, while the latter suggests that every death is premature to some degree. We submit no correct answer here; both seem theoretically plausible, and it is difficult to adjudicate between them. It also does not seem helpful to settle the issue through analogies. For instance, ABTs might suggest a conception of premature death as absolute deprivation, which can be measured by thresholds similar to poverty, while LSDPs might suggest that a relative measure can track the essence of premature death, similar to those used to measure economic inequality. But is premature death, as a phenomenon, more like absolute poverty or economic inequity? Again, we believe that there is no correct answer. It thus seems like conceptual analysis of the term *premature death* or methodological debate cannot help guide choices between conceptions and measures.

Our suggestion, therefore, is that choices between conceptions or measures should be considered on the combined grounds of preferences for normative values and practical issues about the intended application. Certain conceptions reflect specific normative values, as we shall argue, and researchers, policymakers, etc., often have good reasons to choose to reflect such values. As these conceptions are, according to us, on par, we also imply they are not equally well suited to all purposes. Practical issues are thus also important considerations when choosing between conceptions, but exactly how to make such choices or how to balance conflicting normative values is beyond the scope of this article.

### Other Measures

As stated above, we focus on conceptions of premature death based on the dimension of chronological age. However, there are also conceptions based along other dimensions. For instance, the measures of preventable and amenable mortality which classify deaths as premature according to whether they reasonably could have been avoided, regardless of the victims’ ages. Let us call such a dimension preventability. Preventability might also be what is appealed to when certain cause-specific mortality statistics (e.g., tobacco-related deaths) and accidental mortality are purported to measure premature death.^5^

These measures might provide relevant information in settings where premature death is much discussed (e.g., preventable and amenable mortality seem particularly relevant for health system evaluations). But it does not seem like if preventability is necessary for any plausible conception of premature death. From the perspective of population health which we consider, the preventability dimension can be accounted for by, for instance, the study design and thus need not be built into the conception. On the other hand, chronological age seems more indispensable from our considered perspective and is more difficult for the study design to account for after the fact. See Table 2 below for an overview of the purported premature death measures﻿.

**Table ﻿2:** Overview of purported premature death measures

Measure	Measurand	Use	Valid?
1. Age-based thresholds	Number of premature deaths as opposed to non-premature deaths	(i), (ii), (v),	(y)
2. Years of potential life lost (YPLL)	Gross loss of life years due to premature death (determined by an age-based threshold)	(i)–(v)	(y)
3. Years of life lost (YLL) in Disability-adjusted life-years (DALY) metric	Gross loss of life years compared to an ideal (society's) life expectancy	(i)–(v)	(y)
4. Preventable mortality	Number of deaths that could be avoided through large-scale public health policy interventions	(i), (ii), (v)	(n)
5. Amenable mortality	Number of deaths that could be avoided through optimal health care	(i), (ii), (v)	(n)
6. Cause-specific mortality (e.g., tobacco-related deaths)	Number of deaths due to causes that by inference makes the death premature	(i), (ii)	(n)
7. Accidental mortality	Number of deaths due to accidental injury	(i), (ii)	(n)

Overview of purported premature death measures by what they measure (*measurand*), common applications (*use*) and our verdict of their ability to actually track premature death as we shall understand the concept in this article (*valid?*). The common applications are: (i) evaluating public health policies after implementation, (ii) identifying targets for policy interventions, (iii) ranking policy interventions before implementation (e.g., cost-effectiveness analysis), (iv) monitoring mortality trends, and (v) evaluating health systems. In this article, we focus on rows 1–3. Rows 4–7 are mentioned for giving a complete overview and further discussion of these fall outside the scope of this articl﻿e

## Conceptual Issues

As a result of the vagueness of the term *premature death*, the concept has been measured in a wide range of ways. The research literature shows signs of being operationalist. In the philosophy of science, there are two main accounts for the relationship between concepts and their corresponding measures. Realists hold that measures should track objective properties of a concept, whereas nominalists take measures to be definitive of a concept [17]. The current practice in disciplines that study premature death is arguably nominalist. Moreover, that practice seems to coincide with operationalism—the extreme version of nominalism—according to which a measurement operation fully specifies the concept [17]. Operationalism, though generally accepted as an approach to measurement—in particular towards a *Ballung concept* which lacks clear boundaries [18]—might lead to problems of which shall review three: arbitrariness, usefulness, and normative validity.

### Arbitrariness

Complaining about arbitrary thresholds is common in critiques of ABTs (see, e.g., [19]). To elaborate, Martinez et al. [19] argue that AGLNs are preferable over ABTs for monitoring premature death from non-communicable diseases because arbitrary thresholds lack methodological justification and exclude older populations. However, to our knowledge, the charge of arbitrariness has yet to be substantiated. One interpretation of the critique is that there is no rationally compelling reason to set a particular ABT somewhere (e.g., ≤ 65) rather than somewhere else (e.g., ≤ 75). However, this interpretation appears to be false. ABTs are usually informed by life expectancy statistics in some fashion. This implies that a range of ABTs, depending on their purpose, could be considered acceptable—though there might not be a way to settle disputes about whether the placement of a particular ABT within this range is optimal.^6^

Another interpretation of the critique is that ABTs are vague. Though the threshold is supposed to mark whether (and to which degree) a death is premature, no precise degrees of premature death seem to fix the threshold anywhere specific. For example, for any particular ABT (e.g., ≤ 70), there will be borderline cases of nearly premature deaths (e.g., death at 71 years) whose exclusion seems arbitrary, at least compared with the inclusions of those who died just before their 71st birthday, and so on. However, this sharp delineation appears too implausible. It is difficult to consider our intuitive judgment about excluding borderline cases as arbitrary as a disadvantage for ABTs without also assuming that this conception of premature death is incorrect. Given that multiple conceptions of premature death are on par, and ABTs define the term as to apply to deaths below an upper age cut-off; then what is the alternative?

Theoretical disagreements between proponents of ABTs and AGLNs (or LSDPs) can boil down to disputes over the correct conception of premature death. However, such disputes are difficult to resolve by discussing operationalization and methodology. Since the concept of premature death is so slippery, it is not clear whether any of the three categories of measures are theoretically preferable over the others. Thus, in a technical sense, Martinez et al. [19] beg the question against ABTs when claiming that these measures are wrong to exclude older populations, which ABTs do by definition. However, we suspect their claim is ultimately about the measure's practical advantages and disadvantages rather than theoretical ones. After all, Martinez et al. claim that AGLNs are preferable over ABTs for the specific purpose of monitoring premature death from non-communicable diseases. They may well be right about that. But the reasons why will stem from practical details—e.g., who will use information gathered and for what purpose? Thus, many of the issues that arise on this practical level will have a normative dimension.

### Usefulness

Premature death measures are intended for a different albeit partly overlapping set of purposes. ABTs, for instance, are simple and easy to understand. They can thus be useful in institutional settings where the success of an initiative depends, in part, on broad support from different actors. However, ABTs might be inappropriate in some settings, as exemplified by the critique from Martinez et al. above. The AGLN and LSDP measures, on the other hand, can be complex and challenging to understand.

A potential worry, then, is that AGLNs and LSDPs might be impractical for policymakers. As argued by Schroeder, researchers should structure measures to reflect the normative values that policy makers want them to have [23]. However, in the case of AGLNs and LSDPs, this can be hard to achieve in practice since it is not always easy to measure such normative values and often difficult to change the measures after the fact as to reflect them. Adjusting measures in this way requires access to raw data and require calculations that often will be too complicated for policy makers to perform on their own without assistance from researchers [23]. Another related problem comes from the relative similarity of measures of lifespan disparity in LSDPs, which applies to *e*^*†*^, in particular. This is because different lifespan disparity measures generate similar results, but are, in diffuse ways, dissimilar as to which parts of a population's deaths they are most sensitive to changes for [24]. Our proposed approach which emphasizes justification throughout the process of choosing and structuring measures, may help alleviate some of these issues.

### Normative Validity

How can we tell whether a measure of premature death really tracks what premature death is about? This question, which is fundamentally about construct validity, gets more complex if—as we have suggested is the case with premature death measures—the overall approach to measurement is operationalist. One suggested route for such approaches is to consider a construct valid if it coheres with theoretical and empirical knowledge about the concept in question. An issue with operational approaches in certain disciplines is that these focus on empirical knowledge and avoid theory pertaining to the concepts, thus often failing to acknowledge that some concepts are normative [12]. While epidemiology and similar disciplines have not spilt much ink about the validity of premature death measures, there seems to have been some acknowledgement that premature death—or the larger framework which it is part of—is value-laden; see, e.g., [2].

However, it also seems like some effort is put into limiting the extent to which it is acknowledged that such measures are value-laden [25]. We argue that, specifically concerning a notion such as premature death, value-ladenness is problematic as the main issue with premature death measures is their low normative validity. Adapted from Alexandrova and Haybron [12], we propose that the normative validity of a measure of premature mortality should be assessed by the extent to which it aligns with the importance of premature mortality for a legitimate normative goal. Legitimate normative goals, we suggest further, are best grounded in plausible normative theories.

## Normative Background Theories

How can philosophical theorizing assist operationally defined measures? One direction this assistance can take, which is quite common in bioethics, and otherwise suggested by Anna Alexandrova in connection to measurement in general, is to create so-called *mid-level theories*. [26] Mid-level theories are philosophical theories adapted to a relevant context. In this case, the context of justifying conceptions and measures. As such, this article can be understood as answering calls from epidemiologists for more theorizing about the discipline's concepts and value-laden assumptions; see, e.g., [27, 28].

**Fig. 2:**
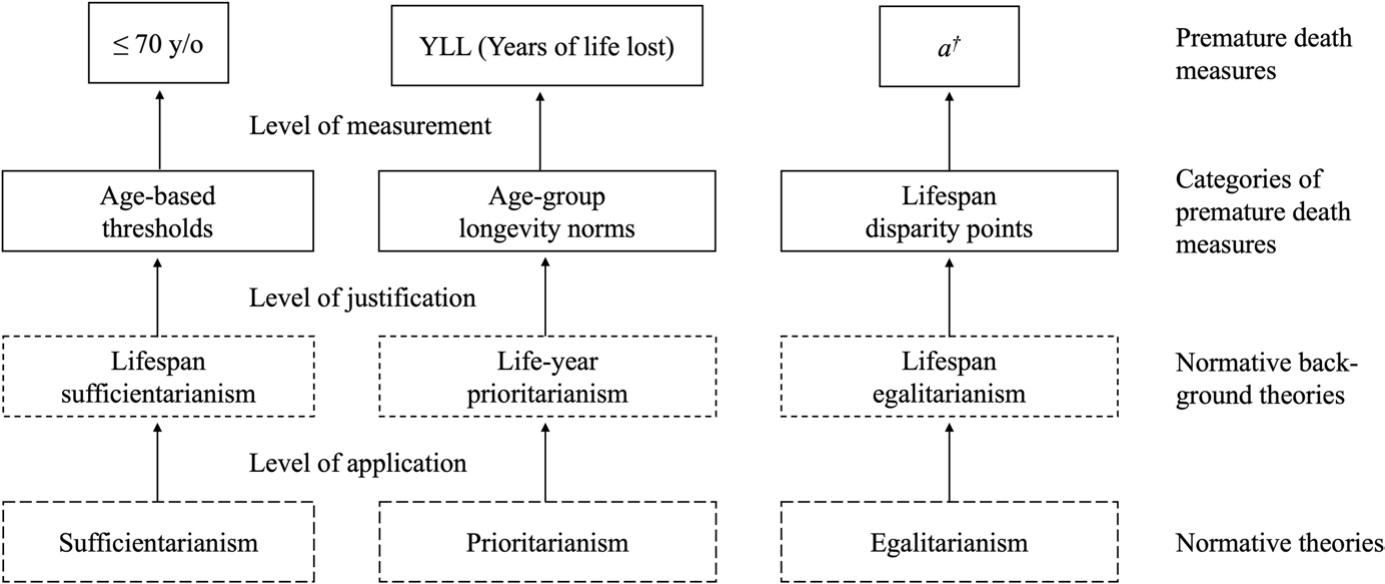
Illustration of the connection between the elements in our framework: Premature death measures are comprised in categories of such measures. The categories can be justified by normative background theories, which are inspired by normative theories applied to the particular setting of measuring premature death. The figure is drawn and adapted from Anna Alexandrova's work on welfare measurement [26, p. XL]

However, our proposal is that philosophical theories remain in the background, rather than on the level of abstraction for the premature death conceptions. We thus dub our adapted theories as "normative background theories". There are, roughly, three levels on which one can attempt to understand current practices in epidemiology and similar disciplines. On the descriptive level, one attempts to understand *what* epidemiologists do (e.g., how they measure premature death.) On the interpretive level, one attempts to understand *why* they do what they do (e.g., why they measure premature death in one way rather than another.) On the normative level, one attempts to say what they *ought* to do (e.g., how they should measure premature death.) While we, in this article, focus on the normative level, we intend to operate at the other levels as well. Our proposal should thus be understood as leaving the existing conceptions and measures much as they are while presenting a menu of normative background theories that can be used to provide additional justification (for an illustration of our framework, see Fig. 2).

There are several plausible normative background theories that could provide theoretical justification for measures of premature death. In what follows, we introduce four plausible candidates, which we then try to adapt to a setting of premature death, life expectancy, and longevity. These are, respectively, *lifespan sufficientarianism*, *age-weighted prioritarianism*, *the harm of death approach*, and *lifespan* egalitarianism. The first, second and fourth candidates are theories about distributive justice, which provide systematic explanations for principles according to which burdens and benefits ought to be distributed [29]. The third candidate theory is value-theoretical: it provides systematic explanations for why the things that are good or bad for a person are, *in fact*, good or bad for them [30]. We shall argue that ABTs can be paired with lifespan sufficientiarianism, AGLNs with the harm of death approach and age-weighted prioritarianism, and LDSPs with lifespan egalitarianism (for an overview, see Fig. 2, and Table 3 below)﻿.

**Table 3:** Categories of premature death measures

Category	Measure	Measurand	Conception	NBT
Age-based thresholds (ABTs)	70 years (UN 2017)	Number of premature deaths as opposed to non-premature deaths	Insufficient length of life	Lifespan sufficientarianism
Age-group longevity norms (AGLNs)	Years of life lost (YLL) in GBD study (Murray 1996)	Total loss of life years compared to an ideal (society's) life expectancy	Gap in mortality between actual and ideal conditions	Harm of death, lifespan prioritarianism
Lifespan disparity points (LSDPs)	e^†^ (“e-dagger”) (Zhang & Vaupel 2009)	Expected disparity of expected remaining life years within populations	Lifespan inequality	Lifespan egalitarianism

For each category we give an example of a measure and its measurand with their implied conception of premature death and suggest which normative background theories (NBTs) they should be paired with

### Lifespan Sufficiency

ABTs require a normative background theory can that justify the practice of setting thresholds for premature death.^7^*Sufficientarianism* is a distributive theory that, very roughly, holds that what matters is not inequality but that everybody has enough of whatever is normatively significant, such as, for example, welfare, resources, and health. To work out what “enough” is, sufficientarianism specifies a so-called *sufficiency threshold* below which persons are considered worse off in an absolute sense. This sense may not be wholly unlike the sense in which ABTs classify deaths under an upper age cut-off as 'premature'. In the context of premature death, we can specify a sufficiency threshold thus:

*Lifespan sufficiency*: There is a certain length of life that is sufficient for anyone.

This specification is not unreasonable. Many philosophers propose sufficiency thresholds based on judgments about a sufficient length of life. For instance, Roger Crisp suggests, “my own intuition is that, say, eighty years of high-quality life on this planet is enough, and plausibly more than enough, for any being”[32]. On Martha Nussbaum’s list of central human functional capabilities, the first item is “[b]eing able to live to the end of a human life of normal length; not dying prematurely”[33]. Also, Madison Powers and Ruth Faden claim that a minimal requirement of sufficiency is that everyone “have enough health over a long enough life span to live a decent life”[34].

A potential issue is that the notion of a sufficient length of life may conflict with the spirit of sufficientarianism. Sufficientarianism is not supposed to place any normative significance on distributive inequality. But judgments about a certain length of life being enough seems comparative rather than absolute—i.e., based on some distribution of lifespans such as the current one.^8^ And if sufficientarianism does not care about inequality, it should perhaps not use any distribution of lifespans as a basis for fixing the sufficiency threshold. It should, instead, use other non-comparative considerations. Here, a potential workaround might be to continuously update the thresholds as death patterns change and mainly employ them at (national or regional) local levels. Though setting threshold is a staple of sufficientarianism, few sufficientarians have attempted to develop these and there is little literature on the rules of their formation, structure or content; see, e.g., [35].

The most important issue is arguably whether a notion of lifespan sufficiency fits as part of a fully specified sufficientarian theory. Sufficientarian theories take on many forms, e.g., basic needs, welfare recommended by an impartial spectator, and required capabilities for dignity or as equal citizens [35]. And it seems plausible that lifespan sufficiency since health or longevity (or whatever else an ABT could be said to measure) seems to matter for sufficiency, however it is construed.

### Age-Weighted Prioritarianism

One would not want to justify a conception of premature death through a health maximization view as fairness—which is a primary concern behind the idea of premature death—does not matter on such views. However, one might want a normative background theory that combines the rationales of maximization and fairness. One such approach is prioritarianism, which assigns more weight to fixed improvements by how much worse off the recipients are [36]. Adapted to the context of population health with emphasis on longevity, prioritarianism would take an age-weighted approach and assign more weight to life years gained the younger the recipients are. Age-weighted prioritarianism seems like an ideal candidate background theory for AGLNs.

The main issue in AGLNs is to specify longevity norms. AGLNs quantifies the total gap in longevity between a particular death and the expected longevity with respect to a reference population by calculating the remaining life expectancy at different ages. This gap constitutes a counterfactual for how much is lost when persons in the population die. Somewhat simplified, there are two main aims in the AGLN literature for calculating remaining life expectancy: *realistic life expectancy* uses local death rates to calculate what is lost by death, whereas *ideal life expectancy*^9^ uses death rates selected from the most long-lived populations in the world [23]. When used as a conception of premature death, the former might suggest a maximizing background theory which only considers the unweighted life years lost, while the latter suggests a prioritarian background theory which assigns more weight to life years lost the younger the victims are. Thus, prioritarianism, in the age-weighted form, would justify AGLNs with ideal life expectancy.

An example of such a measure is years of life lost (YLLs) in the disability-adjusted life year (DALY) framework. Christopher Murray, the chief DALY architect, discusses the approach by way of an example:

[realistic life expectancy] would lead us to conclude that the death of a 40-year-old woman in Kigali contributes less to the global burden of disease than the death of a 40 year-old woman in Paris because the expectation of life at age 40 is lower in Rwanda than in France. Equivalent health outcomes would be a greater burden in richer communities than in poorer communities [2].

Murray states that he has an egalitarian motivation for choosing ideal instead of realistic life expectancy in the YLL [34, p. 15﻿]. However, Murray’s chooice of ideal life expectancy could also be justified by prioritarianism: not choosing ideal life expectancy would effectively disguise absolute worse-offness.

While age-weighted prioritarianism seems like an appropriate background theory for AGLNs relying on ideal life expectancy, AGLNs are still vulnerable to the accusation of arbitrariness. The AGLN-approach to premature death identifies and quantifies a counterfactual loss and prescribes greater priority to larger gaps. However, the AGLN-approach needs to explain why we should prioritize this gap rather than another. For instance, critics of ideal life expectancy have emphasized that it is less informative about local conditions and thus yields less feasible benchmarks for interventions [37]. On the other hand, Hirose has defended ideal life expectancy by suggesting that the YLL’s conception of premature death is not completely arbitrary, since “[t]here is no biological reason for believing that Japanese women should live longer than any other group in the world” [38].

Yet, AGLNs involves setting longevity norms somewhere. Age-weighted prioritarianism is thus well-positioned to provide additional justification for setting norms according to ideal life expectancy justified as giving priority to the worst-off, while realistic life expectancy lacks such additional justification.

### Decreasing the Harm of Death

The *harm of death* debate in philosophy concerns whether—and, if so, to which extent—death harms the person who dies.^10^ If we assume with most philosophers participating in the debate that death can be harmful to those who die, we can use views from this debate as normative background theories. *Deprivationism*, the most basic account of why and how death harms, holds that death is harmful to a person to the extent that it deprives them of intrinsic goods they otherwise would have had; see, e.g., [6, 40, 41]. Deprivationism thus envisages death as an extrinsic, comparative harm; it is harmful not in itself but in terms of its instrumental effects and only to the extent that further life would have been intrinsically good. As a normative background theory, deprivationism could justify initiatives to prevent premature death on broad, welfarist grounds—for instance, as a type of harm reduction.^11^

However, deprivationism is not easily applied to the context of population health. In contrast with the distributive views discussed elsewhere in the article, deprivationism does not consider longevity (e.g., measured as average life expectancy) at the population-level as relevant when evaluating whether and the extent to which death harms a person. This is because deprivationism uses an individualistic approach to answer the counterfactual of what would have happened if the deceased did not die when they did, namely *the nearest possible worlds framework* [42]. On this framework, roughly, we are supposed to consider the further life of the deceased in the nearest possible world holding constant everything except for their specific cause of death.

For instance, if a 70-year-old dies from a given complication of cancer, we cannot abstract away the cancer itself but only that specific, lethal complication [6], which means that the 70-year-old would likely die from another complication not long after. This is in contrast with the actuarial approach to counterfactuals found in epidemiology and similar disciplines, which would ascribe to that 70-year-old the average remaining life expectancy at that age. Deprivationism is thus *not* concerned with age in itself: for anyone for whom death is harmful, death can be worse in all relevant respects for someone who is older.

It is, however, unclear to which extent deprivationism is bound to such principled evaluations and its individualistic approach to counterfactuals. If deprivationism recognizes, as it probably should, the average or aggregate judgment that younger persons lose more further life by dying than older persons do, deprivationism should be able to function as a background theory. In particular, it should fit with AGLNs which are sensitive to the degree of prematurity in death. However, when it comes to life tables, deprivationism would be concerned with the averages of individual losses of further life and thus demand realism in longevity norms, which would justify AGLNs with realistic but not ideal life expectancy.

### Lifespan Egalitarianism

LSDPs define premature death in terms of lifespan inequality. This category of measures uses disparity points to separate the deaths that increase lifespan inequality from the deaths that decrease it. The deaths that increase inequality are designated as the premature ones. LSDPs could therefore draw on egalitarianism as a normative background theory. Egalitarianism is fundamentally concerned with decreasing inequality and increasing welfare. These concerns seem congruent with LSDPs: averting premature deaths—deaths which by definition increase inequality—necessarily transfer these deaths over the disparity point so that they now decrease inequality. Moreover, averting premature deaths likely also increase welfare—at least indirectly, since longer lives generally contain more welfare.

However, beyond this basic congruence, it is not straightforward to pair up LSDPs with egalitarianism. One set of issues stem from equality being a *contested* concept. The first issue is that many non-egalitarian theories are committed to *some* notion of equality. LSDPs should thus reflect a *distinctly* egalitarian notion of equality in order to rely on egalitarianism as a background theory. The second issue is that equality being contested results in several competing versions of egalitarianism. LSDPs should thus accord with at least one of these versions. Another set of issues stem from *inequality* being an elusive concept. Many empirical approaches to measuring inequality are thus thought to fail to capture what inequality truly is; see, e.g., [43, 44]. Whether this is the case with LSDPs remains to be seen. However, we will not impose stricter standards on LSDP than other inequality measures. We will briefly review these issues below.

First, which notion of equality is distinctly egalitarian? An obvious candidate is the comparative notion of equality, which concerns how people fare compared to others [43]. LSDPs might be said—somewhat superficially—to reflect this notion: the disparity point is a 'moving target' dependent on the longevity of a population to which one is compared. Dying prematurely—i.e., below the disparity point—could thus be interpreted as an instance of being worse off *compared* to others.

Second, how does LSDPs accord with different versions of egalitarianism? The two most discussed versions are luck egalitarianism and relational egalitarianism. Somewhat simplified, luck egalitarianism is concerned with compensating individuals that suffer from bad luck, see, e.g., [45], while relational egalitarianism is concerned with citizen's abilities to relate to each other as equals in society, see, e.g., [46]. As far as we know, lifespan inequality or premature death is not discussed in the literature on luck and relational egalitarianism. However, a provisional case could be made for both views being concerned about lifespan inequality.

Proponents of both views are concerned with inequalities in health. For instance, luck egalitarians take many instances of poor health to constitute bad luck. Complications arise over the view's emphasis on personal responsibility, as luck egalitarianism seems to imply that individuals should suffer the consequence of their irresponsible choices [46]. Luck egalitarianism is bound to place *some* importance on personal responsibility for health. However, it is not clear whether, or to which extent, this extends to lifespan inequality. After all, the total length of our lives is largely outside our personal control. An egalitarian background theory to LSDPs should thus be allowed to set this complication of personal responsibility aside. The challenge with relational egalitarianism, on the other hand, is that this view is not clear about the *extent* to which it opposes health inequalities [47]. However, relational egalitarianism has particular grounds to oppose lifespan inequality. It is the length of one's lifespan that determines the extent of one's ability to participate in society over time, e.g., through voting. In a society with too much lifespan inequality, the equal social participation of citizens is compromised.

## Conclusion

This article set out to argue for an approach to justifying existing conceptions and measures of premature death that are becoming increasingly important as practical goals in global health. We have argued that many measures of premature death have low *normative validity*. Moreover, we have proposed that normative background theories could raise the normative validity of these measures by linking them to legitimate normative goals. We have shown that the harm of death approach and prioritarianism could do this for AGLNs, sufficientarianism for ABTs, and egalitarianism for LSDPs. By doing this, the article aimed to raise the legitimacy of these measures and assist with their further use in epidemiology and other disciplines﻿.

## Footnotes

1 We take 'premature death' and 'premature mortality' to have the same meaning.

2 A superficial summary might say that there are few detailed discussions of the concept in epidemiology and other population health disciplines but cursory discussions in philosophy. However, in philosophical discussions, the concept is usually either considered prima facie normatively significant (e.g., [5]) or discarded as too arbitrary to be normatively significant (e.g., [6]).

3 We borrow and develop this concept from Alexandrova and Haybron [12].

4 Note that *remaining expected life-years* here presupposes knowledge of the individual's context's force of mortality paired with a notion of central tendency or the like. *Remaining expected life-years* is also known as *mean residual life* and will be strictly positive unless there is an age beyond which no-one survives. Note further that one could also employ e.g., *median* residual life, or other measures of averages or central tendency.

5 Some cause-specific and accidental mortality statistics also seems to appeal to other dimensions, such as the distinction between natural and non-natural death and internal and external causes of death.

6 Conventions could diffuse such disputes, though they would arguably do so without providing rationally compelling reasons for choosing any particular ABT. Arbitrary thresholding is abundant in science. Individual instances of the practice are not obviously illegitimate or inferior, even if alternatives that do not rely on arbitrary thresholds exist. This seems to hold for quantitative studies of social phenomena, which often involve operationalizations of *Ballung* concepts. We suspect that the charge of arbitrariness might lose some of its appeal (and rightfully so) if appropriate bodies managed to develop acceptable standards or guidelines for how life expectancy statistics should inform the practice of setting ABTs. However, in the case of premature mortality, this is easier said than done: researchers have suggested conventions for setting ABTs since, at least, the inception of *potential years of life lost* (PYLL) measure in the 1940s (see, e.g., [20–22]).

7 The fair innings view might be another candidate. However, it is a primarily a view about priority setting in health and, as such, it requires a philosophical defense on its own [31].

8 For example, Crisp's intuition about 80 years as enough would lose its appeal if, say, the global average life expectancy at birth was to significantly increase at some point in the future. Nussbaum's notion of a “life of normal length,” if it relies on life expectancy statistics (which is plausible), would lose its appeal given significant increases in lifespan inequality.

9 Schroeder distinguishes between aspirational and counterfactual life expectancy, which we rename as ideal and realistic life expectancy [23].

10 The connection between this philosophical debate and priority setting in health is explored in [39].

11 Here we mean *harm reduction* in a generic sense and not the liberal approach to certain self-harming acts often associated with drug policy﻿.
